# Depression Among Individuals with Irritable Bowel Syndrome: A Nationwide Claims-Based Analysis of 3.9 Million Koreans

**DOI:** 10.3390/healthcare13232998

**Published:** 2025-11-21

**Authors:** Yongjoo Kim, Dongsu Kim, Hanbit Jin, Eunji Ahn, Rachel F. Rodgers, Eric Bui, Gihyun Lee, Jae-Hong Kim, Young-Ho Moon, Kyeong-Ok Kim, Taewon Kim, Mee-Hyun Lee

**Affiliations:** 1 Department of Korean Medical Sciences, College of Korean Medicine, Sangji University, Wonju 36686, Republic of Korea; yongjoo.kim@mail.harvard.edu (Y.K.); taewonkim1580@gmail.com (T.K.); 2 Department of Preventive Medicine, College of Korean Medicine, Dongshin University, Naju 58245, Republic of Korea; dskim20@dsu.ac.kr (D.K.); hanbitjin22@gmail.com (H.J.); ahneunji1015@daum.net (E.A.); 3 Department of Applied Psychology, Northeastern University, Boston, MA 02115, USA; r.rodgers@northeastern.edu; 4 Unité de Recherche Clinique et d’Innovation, Hôpital du Cotentin, 50100 Cherbourg, France; 5 Department of Psychiatric Emergency & Acute Care, Lapeyronie Hospital, CHRU Montpellier, 34090 Montpellier, France; 6 Department of Psychiatry, Caen University Hospital, Caen University, 14000 Caen, France; bui-th@chu-caen.fr; 7 Korean Medicine Research Center for Bi-Wi Control Based Gut-Brain System Regulation, College of Korean Medicine, Dongshin University, Naju 58245, Republic of Korea; glee@dsu.ac.kr; 8 Department of Acupuncture and Moxibustion Medicine, Gwangju Korean Medical Hospital, Dongshin University, Gwangju 58245, Republic of Korea; nahonga@dsu.ac.kr; 9 Department of Internal Medicine, Mokpo Korean Medical Hospital, Dongshin University, Mokpo 58245, Republic of Korea; doc4you@dsu.ac.kr; 10 Department of Neuropsychiatry, Gwangjoo Korean Medical Hospital, Dongshin University, Gwangju 58245, Republic of Korea; avecinok@dsu.ac.kr; 11 Department of Herbology & Herbal Formula Science, College of Korean Medicine, Dongshin University, Naju 58245, Republic of Korea

**Keywords:** depression, irritable bowel syndrome (IBS), gut–brain interaction, Korean National Health Insurance Service (NHIS)

## Abstract

**Background/Objectives**: While a growing body of research indicates an increased psychiatric burden, including depression comorbidity, among individuals with irritable bowel syndrome (IBS), evidence from large claim-based databases remains limited. This study aimed to investigate the association between IBS and depression among a nationally representative sample of 3.9 million Korean adults. **Methods**: We used data from the 2021 Korean National Health Insurance Service (NHIS), including a total of 3,864,586 individuals aged 19–64 years who participated in the national biennial health screening exam between 1 January 2021 and 31 December 2021. Exposures and outcomes were identified based on the International Classification of Diseases, Tenth Revision (ICD-10), codes: F32 and F33 for depression and K58 for IBS. Multivariable logistic regression analysis adjusted for age, sex, residential area, comorbidity, smoking, alcohol use, physical activity, and body mass index was conducted. **Results**: One-year prevalence of depression among the entire sample was 4.0%. The prevalence among individuals with IBS (7.3%) was significantly higher than that among those without IBS (3.6%, *p* < 0.001). Multivariable logistic regression analysis confirmed this relationship (OR = 1.77, 95% CI: 1.74, 1.79) after adjusting for all covariates. **Conclusions**: Our findings suggest that individuals with IBS are vulnerable to depression, emphasizing the need for integrated approaches in clinical management and public health policy responding to the rising burden of IBS.

## 1. Introduction

Irritable bowel syndrome (IBS) is a chronic gut–brain disorder characterized by recurrent abdominal pain and altered stool frequency or form [1,2]. Clinical manifestations of IBS can be classified into four subtypes, including constipation-predominant (IBS-C), diarrhea-predominant (IBS-D), mixed (IBS-M), and unclassified (IBS-U) subtypes [3,4,5]. Globally, 9.2% of adults meet the diagnostic criteria of IBS, and a higher prevalence is reported in women (12.0%) than men (8.6%) [5,6]. Beyond gastrointestinal symptoms, IBS is often followed by substantial systemic sequelae such as fatigue, fibromyalgia and chronic pain, and lower urinary tract symptoms over a patient’s lifetime, which can significantly reduce health-related quality of life [7,8,9,10].

In addition to these physical impacts, IBS has been suggested to be implicated in poor mental health, and particularly depression. Indeed, emerging evidence points to the potential role of a bidirectional gut–brain axis in the development of depression among those with IBS. Proposed mechanisms include gut microbiota dysbiosis, hyper-activation of the hypothalamic–pituitary–adrenal (HPA) axis, serotonergic signaling abnormalities, and reduced vagal (parasympathetic) tone [10,11,12,13,14,15].

For instance, individuals with IBS have been found to have decreased diversity in gut microbiota, affecting the production of neuroactive metabolites such as short-chain fatty acids (SCFAs) and tryptophan catabolites, followed with alterations in the signaling of neurotransmitters (e.g., GABA, serotonin, and BDNF), which in turn can lead to increased risk of depression [11,12,15]. Studies have also shown that the increased intestinal permeability often observed in individuals with IBS can facilitate systemic inflammatory process by producing pro-inflammatory cytokines such as IL-6 and TNF-α, which can also contribute to the development of depression [11,12]. Additionally, IBS is often followed by persistent hyper-activation of the HPA axis and down-regulation of gut–brain serotonergic signaling and vagal system function, all of which can in turn lead to elevated risk of depression [11,12,13].

Consistent with this proposed basis for the comorbidity of IBS and depression, empirical work has documented a higher prevalence of psychiatric comorbidities, such as depressive or anxiety symptoms and disorders, among individuals with IBS, compared to the general population [10,16,17,18]. For example, Zamani and colleagues (2019) conducted a meta-analysis of 73 studies and documented that the prevalence of depression and depressive symptoms among those with IBS was 23.3% and 28.8%, respectively, two-to-three-fold greater than among the general population [16]. In an earlier meta-analysis, Fond and colleagues (2014) also reported similar findings, in which individuals with IBS tended to have higher depressive symptoms than healthy controls [17].

The prevalence of IBS among Koreans has been reported to be 6.0–15.6%, depending on the assessment method, which is generally higher than the values recorded in other East Asian countries [18,19]. Moreover, the burden of depression has substantially increased from 2.8% during 1998–2005 to 5.0% in 2020 [20,21]. However, while the IBS–depression associations among Western populations, such as North American, European, and Oceanian populations, are well documented [16,17], evidence among the Korean population has been limited to small or convenience samples that relied on screening tools for depression measurement. For instance, a single-center study of Korean adults (124 IBS outpatients and 91 healthy controls) indicated that 31% of the cases and 16.5% of the controls were classified as having depression, measured with the Hospital Anxiety and Depression Scale [22]. Another study based on the Korea Nurses’ Health Study cohort demonstrated that 13.5% of nurses with IBS (*n* = 178) screened positive for depression, measured with the Patient Health Questionnaire-9 [23]. While these previous studies showed a higher prevalence of depression among those with IBS compared to healthy controls, their findings were based on relatively small-sized samples and self-reported screening tools for depression rather than diagnostic measures, limiting the representativeness and robustness of the findings and thereby necessitating further investigation.

Over the past two decades, the prevalence of depression among Koreans has rapidly increased, nearly doubling from 2.78% in 1998–2005 to 5.06% in 2021 [21]. Moreover, depression has been consistently linked to an increased risk of suicidality among Koreans [24]. Given that the suicide rate in Koreans has been the highest among Organization for Economic Cooperation and Development (OECD) countries over the past two decades [25], understanding the connection between IBS and depression would provide important policy and clinical implications. To more rigorously replicate findings regarding the comorbidity of IBS and depression outside of Western contexts, we sought to investigate the association between IBS and depression among a nationally representative sample of Korean adults. We hypothesized that individuals with IBS would be more likely to be diagnosed with depression than their counterparts without IBS. We used the health screening subset of the 2021 National Health Insurance (NHIS) database, which includes diagnostic information on relevant disorders and health screening exam records for 3.9 million Korean adults, enabling us to understand this relationship in a nationwide setting among the population.

## 2. Materials and Methods

### 2.1. Data Source and Study Sample

Data from the 2021 Korean National Health Insurance Service (NHIS) database were utilized. In South Korea, healthcare utilization is primarily covered by the NHIS, in which all Korean citizens are registered and enrolled. This provides universal coverage for healthcare utilization for 97% of the entire population, together with additional medical aid support for the economically vulnerable subpopulation, which represents approximately 3% of the total population [26,27,28]. In 2021, a total of 51,412,000 Koreans (99.3% of the entire population) were enrolled in the national health insurance program. The NHIS database includes information pertaining to demographics and qualification (e.g., age, sex, residential area, income, and insurance enrollment type), as well as healthcare utilization obtained through the providers’ claims process (e.g., diagnostic, therapeutic, and prescription information covered by national insurance) [26,27,28].

**Figure 1:**
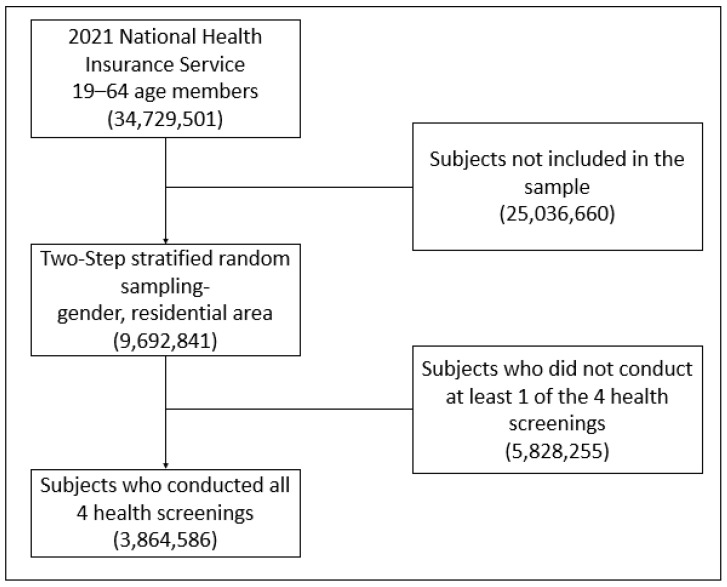
Sample selection flow chart from the 2021 Korean National Health Insurance Service database.

Using the healthcare utilization data from the NHIS database obtained between 1 January 2021 and 31 December 2021, those pertaining to individuals aged between 19 and 64 years were included, identifying 34,729,501 individuals. A sex- and region-stratified two-stage sampling approach was then used, yielding a sample of 9,692,841 individuals. The NHIS provides coverage for a biennial health screening service, in which information on health-related behaviors, anthropometric information, and laboratory results are obtained [26,27,28]. This screening information was used to narrow the sample to individuals for whom information on smoking, drinking alcohol, physical activity, and body mass index was provided. After excluding those with incomplete information for any of those variables, our final sample for the analysis included 3,864,586 participants (Figure 1).

This study used a publicly available and de-identified anonymized dataset and was approved by the Institutional Review Board of Dongshin University (IRB number: 1040708-202212-SB-045).

### 2.2. Measures

#### 2.2.1. Depression

Depression, the outcome of interest in this study, was defined by the International Classification of Disease, Tenth Revision (ICD-10), codes F32.X (depressive episode, including single episode of major or other depressive disorders) or F33.X (recurrent depressive disorder). Depression cases were identified by the presence of any of these diagnostic codes at least once from 1 January 2021 to 31 December 2021 [29,30].

#### 2.2.2. Irritable Bowel Syndrome

Our primary exposure of interest, IBS, was defined by the ICD-10 code K58.X (irritable bowel syndrome and its subtypes). Exposure status was classified as “exposed” when individuals were diagnosed with IBS at least once between 1 January 2021 and 31 December 2021 [31,32,33].

#### 2.2.3. Covariates

A range of sociodemographic, behavioral, and medical conditions were included as covariates. Sociodemographic information included age (in years), sex, and residential area, which were extracted from the individual NHIS records.

To assess overall health condition, the Charlson Comorbidity Index (CCI) [34], a widely used tool to evaluate the overall comorbidity in clinical research, was employed. Based on the 17 prespecified chronic condition groups (e.g., acute myocardial infarction, congestive heart failure, chronic pulmonary disease, dementia), each disease that belongs to the groups is assigned a weight from 1 to 6 (e.g., 2 for cancer without metastasis; 6 for cancer with metastasis). The weighted sum reflects the overall severity of disease burden, with higher scores indicating worse health condition and greater morbidity. The use of ICD-10 codes for calculating the CCI has been validated in previous research [35,36,37]. Based on the guidelines from the NHIS, Quan’s method for calculating participant CCI score was utilized [38], and scores were further classified into three categories: “0” (none), “1” (mild-to-moderate condition), and “2 or above” (severe condition).

Behavioral and metabolic variables such as smoking, alcohol use, physical activity, and body mass index were considered based on previous research showing the association of such factors with IBS [39,40] and/or depression [41,42,43,44]. Standardized self-reported questionnaires were used to measure cigarette smoking, alcohol consumption, and physical activity. To measure smoking behavior, participants were queried on their smoking habits by a two-stage question: (a) the first question asked about smoking status (never smoker, former smoker, and current smoker), while (b) the second question pertained to the duration and amount of smoking for those who identified as former or current smokers. The information was collapsed into two categories: “current smoker” and “former/never smoker” [30].

Alcohol consumption was measured with two questions asking about (a) the average number of days that individuals engage in alcohol drinking per week and (b) the average number of drinks that the individual consume when drinking [45,46]. Based on this information, alcohol use behaviors were classified into two categories: “current drinker” (at least one drinking occasion weekly) and “former/never drinker” (no drinking occasion currently).

Regarding physical activity, participants were asked about the number of days in a week that they engaged in (a) vigorous physical exercise for more than 20 min (e.g., running, cycling) or (b) moderate-level physical exercise for more than 30 min (e.g., jogging) over the past week. Participants who reported engaging in at least one type of physical exercise were considered “physically active” (regular vigorous-to-moderate physical activity), whereas those who reported no days of either type of physical activity were considered as “physically inactive” (no regular vigorous-to-moderate physical activity) [47,48].

Anthropometric assessment was conducted during the biennial health screening check-up by trained staff. Body mass index (BMI) was calculated as weight in kilograms divided by the square of height in meters. According to the guidelines of the Korean Society for the Study of Obesity, we used 25 kg/m^2^ as a cut-off point to define obesity [49]. Since BMI class is considered a covariate, rather than the primary focus of our analysis, we used 20 kg/m^2^ as another cut-off value to group BMI status, resulting in “less than 20 kg/m^2^” (lower BMI group: 10.8% of the sample), “20 kg/m^2^ to 25 kg/m^2^,” (middle range BMI group: 50.0%), and “25 kg/m^2^ or above” (higher BMI group: 39.1%).

### 2.3. Analyses

First, the distribution of the sociodemographic, medical, and behavioral characteristics of the study participants (a total of 3,864,586 individuals aged 19–64 years who went through the biennial health screening exam in 2021) were examined (e.g., means and standard deviations for continuous variables and frequencies and proportions for binary/categorical variables).

Additionally, the 1-year prevalence of depression across the presence or absence of IBS diagnosis in 2021 was established, and a chi-squared test examined potentially differential distribution across a two-by-two contingency table for those variables. To evaluate the extent to which this health screening subset represents the entire population in the 2021 NHIS dataset, we further performed sensitivity analyses among the full eligible sample (*n* = 9,692,841) examining the prevalence of depression by IBS status, regardless of the health screening exam status for that year.

Then, to determine the cross-sectional association between IBS and depression, multivariable logistic regression analysis was performed, with diagnosis of depression as a dependent variable and diagnosis of IBS as a primary exposure variable. To address potential confounding, covariates such as age (in years), sex, residential area (rural vs. urban), CCI category (none, 1, 2 or more), smoking status (active smoker vs. inactive smoker), alcohol drinking status (active drinker vs. inactive drinker), physical activity (physically active vs. inactive status), and BMI class were included. For all analyses, the level of statistical significance was set at 0.05 (two-tailed). All analyses were conducted with SAS version 9.4 (SAS Institute Inc., Cary, NC, USA).

## 3. Results

### 3.1. Sample Characteristics

Table 1 presents the sociodemographic, medical, and behavioral characteristics of the study sample (*n* = 3,864,586). Depression diagnosis was more prevalent in women (vs. men) and among those who were older, lived in a rural area (vs. urban), displayed more comorbid conditions, did not engage in smoking (vs. active smoking), did not use alcohol (vs. active alcohol users), and displayed a low BMI (i.e., <20 kg/m^2^).

This pattern remained generally consistent when performing sensitivity analysis using the full eligible sample regardless of health screening participation (*n* = 9,692,841), showing a greater prevalence of depression diagnosis among women (vs. men) and those who were older, lived in rural areas (vs. urban), and displayed more comorbid conditions (Table S1).

### 3.2. Prevalence of Depression and IBS

Table 2 shows the 1-year prevalence of depression and IBS in the study sample (*n* = 3,864,586). Overall, 4.0% of the study participants were diagnosed with depression and 10.4% with IBS at some point in 2021. However, the 1-year prevalence of depression among individuals with IBS was 7.3%, which was nearly two times that among those without IBS (3.6%; *p*-value < 0.001).

Sensitivity analysis with the full eligible sample regardless of health screening participation (*n* = 9,692,841) showed generally similar results, with a 7.5% of prevalence for IBS and 4.3% for depression among the full sample, as well as 8.5% for depression among those with IBS versus 4.0% for depression among those without IBS (Table S2).

### 3.3. IBS–Depression Association

Table 3 presents the results of the multivariable logistic regression analysis among 3,864,586 study participants who went through the health screening process in 2021. First of all, there was a significant positive association between IBS and depression (OR 1.77, 95% CI: 1.74, 1.79; *p* < 0.001) after adjusting for demographic (e.g., age, sex, and residential area), medical (e.g., CCI), and behavioral characteristics (e.g., smoking, drinking, physical activity, and BMI). That is, individuals who were diagnosed with IBS were 77% more likely to have a depression diagnosis in the same year, compared to their counterparts without IBS.

Additionally, demographic factors such as being a woman (vs. a man) and living in a rural area (vs. urban) were found to be associated with a higher likelihood of having a depression diagnosis, independently of all other covariables. Among behavioral and medical factors, having more comorbid conditions and being an active smoker (vs. former or non-smoker) were associated with a higher likelihood of being diagnosed with depression at some point in the same year, after adjusting for all covariables.

Our sensitivity analysis based on the full eligible sample regardless of health screening participation (*n* = 9,692,841) presented largely consistent results. Compared to those with no diagnosis of IBS, individuals with a diagnosis of IBS in 2021 were more likely to be diagnosed with depression in the same year (OR 1.82, 95% CI: 1.81, 1.84; *p* < 0.001), after adjusting for age, sex, residual area, and health condition (i.e., CCI). Similarly, being a woman (vs. a man), living in a rural area (vs. urban), and having more comorbid conditions was associated with a higher likelihood of being diagnosed with depression, after adjusting for all covariates (Table S3).

## 4. Discussion

The aim of this study was to investigate the cross-sectional association between IBS and depression among 3.9 million Koreans aged 19–65 using the health screening subset of the 2021 Korean National Health Insurance Service (NHIS) database. The one-year prevalence of depression among Koreans with IBS was 7.3%, nearly twice that in those without IBS (3.6%). This pattern remained statistically significant (OR = 1.77, 95% CI: 1.74–1.79) even after adjusting for sociodemographic (e.g., age, sex, residential area), behavioral (e.g., smoking status, alcohol use, physical activity), and medical conditions (e.g., comorbidity index and BMI class).

Our findings are generally in line with prior research, which has evidenced higher prevalences of psychiatric conditions such as depression and anxiety among individuals with IBS [10,16,17]. Our study builds on this incrementally by extending the preliminary findings among Korean individuals [22,23] and providing the first methodologically rigorous test of these associations in a nationally representative sample.

Despite these similar patterns, it should be noted that compared to those previous international and domestic findings, the prevalence of depression reported in our study was relatively low, at 7.3% among those with IBS and 3.6% among those without IBS. The 2021 National Mental Health Survey of Korea reported that the 1-year prevalence of depression assessed with a standardized clinician-administered diagnostic interview among Koreans was 1.7% (men 1.1% and women 2.4%) in a nationally representative sample of 5511 Koreans aged 18–79 years [50]. The discrepancy between the registry-based and community-based statistics in 1-year prevalence of depression (3.6% vs. 1.7%) may be due to the different natures of the databases: (a) a claims-based database, whereby diagnostic/administrative errors or billing-purpose depression coding are common, and (b) structured interviews, whereby social desirability bias or fear of stigma can affect the responses when conducting a person-to-person interview [50,51,52,53].

Moreover, the prevalence of depression in Western populations has been reported to be generally higher than that in Asian populations [54,55,56], which may partly explain the relatively smaller effect sizes found in our analysis compared to those from previous Western studies. According to a recent study by Kang et al. (2023) [21] that utilized self-reported depression diagnosis information from nationally representative surveys of Koreans, there has been an overall increasing trend in the prevalence of depression among Korean adults from 1998 to 2020 (e.g., 2.78% in 1998–2005, 2.99% in 2007–2009, 3.82% in 2010–2012, 3.93% in 2013–2015, 4.14% in 2016–2019, and 4.96% in 2020). However, there was only a slight increase in depression during the COVID-19 pandemic (4.96% in 2020 and 5.06% in 2021) [21]. Another study by Jeong et al. (2020) based on the Patient Health Questionnaire-9 from nationally representative surveys reported similar findings, in which the prevalence of depression among Korean adults was 4.9% during 2016–2018 and 5.3% in 2020 [57]. These findings provide a similar level of depression prevalence to that found in our study.

Prior studies have pointed to the potential role of social and cultural stigma attached to mental healthcare utilization, lack of awareness of depression, and cultural differences in narratives and expressions pertaining to psychological distress (e.g., emphasizing emotional restraint and collective harmony) in shaping these discrepancies in depression prevalences across different cultural contexts or even underestimating the true prevalence of depression at a population level over the past decades [58,59,60,61,62,63,64,65]. Moreover, South Korea has been recognized as being successful in responding to the COVID-19 pandemic, particularly in what is known as the “3T” strategy (Testing–Tracing–Treating), allowing for effective containment with generally lower levels of social restriction and lockdowns compared to many other countries during the period (Hong 2020), which may also explain the relatively lower prevalence of depression in Koreans during the COVID-19 pandemic [66].

We found that women are more likely to be diagnosed with depression than men. This is consistent with previous evidence among Koreans. For instance, Rim et al. (2023) analyzed the 2021 Korean National Mental Health Survey and found that both the lifetime and 1-year prevalences of depression were 1.5- to 2-fold higher among women than men [50]. Similar findings were reported by Lee & Kim (2023), who used the 2020 Korean National Health and Nutrition Examination Survey [59]. The existing literature has documented that the prevalence of internalizing disorders is generally higher among women than men and indicated the significant roles of gender bias and the socio-cultural context, which systematically impose ultimately greater psychosocial burdens on women in South Korea [60,61,62].

The findings from our study present important implications. First, the prevalence of comorbid IBS and depression provide support for explanatory frameworks that emphasize the role of neuroendocrine factors in the etiology of both of these disorders [10,11,12] and the usefulness of pursuing research into the development of mental and physical health concerns from this perspective. Indeed, other work has also highlighted the comorbidity of IBS and anxiety-based disorders, including panic disorders [67,68]. Additional work exploring these gut–brain relationships would be informative. In particular, research focused on understanding the biological, psychological, and sociocultural factors that may predispose, precipitate, and maintain the core elements of both IBS and depressive and anxiety disorders would be useful. Moreover, as noted previously, much of the research in this area is cross-sectional, and rigorously designed studies capable of examining the directionality of the underlying relationships between these disorders would be helpful. Such studies may involve methodologies such as Ecological Momentary Assessment or daily diaries to help capture the sequencing and frequency of physical and emotional experiences among individuals with comorbid IBS and depression [69,70].

In terms of clinical implications, these comorbidities highlight the potential for integrated and holistic interventions for individuals with IBS and comorbid depression. Psychological treatments for IBS grounded in cognitive–behavioral frameworks have previously been shown to be successful in the treatment of IBS, with a focus on cognitive reframing, emotional regulation, and mindfulness techniques [71]. Given the centrality of these same skills in most psychological treatments for depression [72], it is likely that treatments that target the psychological and physiological manifestations of IBS and depression within unified approaches would have a high potential for success. Current first line treatments for IBS include psychoeducation; however, the provision of cognitive and emotional skills is not yet common in clinical practice [73].

However, the findings of our study should be interpreted in consideration of several limitations. First, the cross-sectional nature of our study does not allow us to disentangle the temporality between key variables and thus limits causal inference, particularly susceptible to reverse causation and unmeasured confounding. The existing literature points to a bidirectional relationship between IBS and depression, providing evidence for both IBS-to-depression and depression-to-IBS pathways [74,75]. However, given the dearth of population-level evidence regarding the overarching association between IBS and depression in the Korean context, our current study utilized a cross-sectional design primarily to identify this overall association using a nationally representative claims-based dataset. This warrants future prospective research among Koreans, further elucidating the potential bidirectionality of the relationship. Second, previous studies have documented evidence of an association of diet (e.g., ultra-processed foods) and infection (e.g., COVID-19) with IBS and depression, respectively, suggesting potential confounding [76,77,78,79]. However, in our study, since the NHIS did not collect information on dietary behaviors, we were not able to consider the potential role of diet in our analysis. Further prospective studies with more comprehensive measures of potential confounding variables are warranted to draw causal inference. Third, while previous studies documented other psychiatric conditions, such as anxiety, as other psychological comorbid conditions of IBS, we focused on drawing a nationally representative IBS–depression association among Koreans. Future research may benefit from extending the scope of psychiatric conditions among individuals with IBS to a wider group of mental disorders or further elucidating potential roles of other psychiatric conditions in the IBS–depression relationship. Fourth, we classified smoking status as current vs. former/never smokers, collapsing never smokers and former smokers into one category. However, a recent study documented that, compared to heavy continuous smokers, the risk of developing depression was significantly decreased among never smokers (HR 0.82, 95% CI 0.70–0.97) and long-term quitters (HR 0.69, 95% CI 0.56–0.85), while there was no significant difference when comparing short-term quitters vs. heavy continuous smokers (e.g., HR 0.78, 95% CI 0.50–1.23) [80]. Despite the overall protective directionality of the point estimators, this may lead to insufficient adjustment of confounding by smoking status. However, since our study was based on a cross-sectional design, we were technically unable to distinguish between long-term and short-term quitting status, requiring further investigation with more refined classification of smoking status. Similarly, further research with more refined assessment and classification of other confounders, such as alcohol drinking and physical activity status, is needed. For instance, in our study, we classified physical activity as a physically active status (e.g., at least one or more instances of moderate-to-vigorous physical activity) versus inactive status. While this approach roughly incorporates the current guidelines of physical activity for IBS patients (e.g., 3-to-5 times of moderate-to-vigorous physical activity weekly), it is susceptible to misclassification, such that some portion of individuals who were classified as physically active (e.g., once or twice weekly) may not actually reach the required level of physical activity based on the existing evidence [81,82]. Furthermore, our study did not include information on various other forms of physical activity, such as yoga, which has been known to be associated with improved physical and psychological conditions in IBS patients [83]. Lastly, our study did not examine any potential interactions by IBS subtype, sociodemographic information, or health-related behaviors. While our analysis provides nationally representative estimates of the association, future research should elucidate potential sources of heterogeneity across biological, clinical, and behavioral dimensions.

Nonetheless, our study has several strengths. First, we used a nationally representative sample of 3.9 million Koreans based on the NHIS database to improve the extent of the representativeness of our analysis, thereby providing a sufficient level of external validity of the findings to Korean populations. Second, the claims-based nature of the NHIS database enabled us to utilize physician-driven diagnostic decision information on the core variables, providing a more robust research context to our investigation of the IBS–depression association, pertaining to the validity of the measure, compared to the use of screeners. Third, since we used a health screening subset of the 2021 NHIS database, we were able to obtain information on the behavioral characteristics and the sociodemographic and medical conditions of the participants and adjust for those factors in our multivariable logistic regression model. Thus, the observed IBS–depression connection in our analysis goes beyond the potential confounding or mediating role of such factors.

## 5. Conclusions

In conclusion, in a large, nationally representative sample of Korean adults, we found that IBS was associated with a 77% increased risk for depression in the same year, after controlling for sociodemographic, behavioral, and other medical conditions. Our findings warrant the need for integrated approaches to prevent and address the growing psychiatric burden that individuals living with IBS suffer from. More specifically, public health policies that encourage and support timely screening for depression and referral to psychiatric or psychological specialists, when necessary, should be considered in clinical practice [84]. Last but not least, further studies that can more precisely disentangle the subgroups across sociodemographic, biological, or medical conditions that are at greater risk would be warranted.

## Figures and Tables

**Table 1:** Characteristics of study participants: Korean adults aged 19–64 years from 2021 NHIS database who completed health screening exam (*n* = 3,864,586).

Variables	Diagnosis of Depression in 2021 *n* (%)		
	No Depression (*n* = 3,711,057)	Depression (*n* = 153,529)	*p*-Value
**Age (mean** **± SD)**	44.83 ± 11.88	46.57 ± 12.49	<0.001
**Sex**			
Male	1,945,484 (97.1)	57,741 (2.9)	<0.001
Female	1,765,573 (94.9)	95,788 (5.2)	
**Residential area**			
Rural	1,624,257 (96.0)	68,306 (4.0)	<0.001
Urban	2,086,800 (96.1)	85,223 (3.9)	
**CCI**			
0	2,399,874 (97.3)	69,397 (2.8)	<0.001
1	796,377 (95.0)	41,768 (5.0)	
2+	514,806 (92.4)	42,364 (7.6)	
**Smoking**			
Former/never smoker	2,914,927 (95.9)	124,004 (4.1)	<0.001
Current smoker	796,130 (96.4)	29,525 (3.6)	
**Drinking**			
Former/never drinker	2,032,710 (95.5)	96,460 (4.5)	<0.001
Current drinker	1,678,347 (96.7)	57,069 (3.3)	
**Moderate-to-vigorous physical activity**			
No	1,929,943 (96.3)	74,931 (3.7)	<0.001
Yes	1,781,114 (95.8)	78,598 (4.2)	
**BMI (kg/m^2^)**			
Under 20	397,317 (95.1)	20,674 (5.0)	<0.001
20–25	1,857,589 (96.1)	76,063 (3.9)	
Over 25	1,456,151 (96.3)	56,792 (3.8)	

NHIS, National Health Insurance Service; CCI, Charlson Comorbidity Index; BMI, body mass index.

**Table 2:** One-year prevalence of depression among study participants (*n* = 3,864,586) with and without irritable bowel syndrome in 2021.

		Depression Diagnosis in 2021		Total *n* (%)	*p*-Value
		None *n* (%)	Yes *n* (%)		
**IBS diagnosis in 2021**	**None**	3,337,195 (96.4%)	123,964 (3.6%)	3,461,159 (100%)	<0.001
	**Yes**	373,862 (92.7%)	29,565 (7.3%)	403,427 (100%)	
**Total**		3,711,057 (96.0%)	153,529 (4.0%)	3,864,586 (100%)	

IBS, irritable bowel syndrome.

**Table 3:** Cross-sectional multivariable logistic regression model analyzing the association between irritable bowel syndrome and depression among the study participants (*n* = 3,864,586).

Variables	Values	OR (95% CI)	*p*-Value
IBS	No	Reference	
	Yes	1.77 (1.743, 1.79)	<0.001
Age (years)		1.00 (1.00, 1.00)	<0.001
Sex	Male	Reference	
	Female	1.82 (1.80, 1.85)	<0.001
Residential Area	Rural	Reference	
	Urban	0.99 (0.98, 1.00)	<0.001
CCI	0	Reference	
	1	1.73 (1.71, 1.75)	<0.001
	2+	2.63 (2.60, 2.67)	<0.001
Smoking	Former/never smoker	Reference	
	Current smoker	1.29 (1.27, 1.31)	<0.001
Drinking	Former/never drinker	Reference	
	Current drinker	0.87 (0.86, 0.89)	<0.001
Moderate-to-vigorous physical activity	Physically inactive	Reference	
	Physically active	1.09 (1.078, 1.1)	<0.001
BMI (kg/m^2^)	<20	Reference	
	20–25	0.95 (0.944, 0.96)	<0.001
	25+	0.94 (0.94, 0.957)	<0.001

IBS, irritable bowel syndrome; CCI, Charlson Comorbidity Index; BMI, body mass index.

## Data Availability

The data used in this study are available from the Korean National Health Insurance Service (NHIS: http://nhiss.nhis.or.kr). Access to the data for this study was granted under an NHIS license (NHIS-2024-1-500) and accessed from 1 August 2024 to 31 January 2025, and is therefore not publicly available. Researchers may apply for access by submitting a data request application to the NHIS, meeting the eligibility criteria, obtaining approval from the NHIS review committees, and paying the required data access fees.

## Supplementary Materials

The following supporting information can be downloaded at https://www.mdpi.com/article/10.3390/healthcare13232998/s1, Table S1: Characteristics of all eligible individuals aged 19–64 years from the 2021 NHIS database (*n* = 9,692,841), regardless of their participation in health screening exams; Table S2: One-year prevalence of depression among all eligible individuals aged 19–64 years from the 2021 NHIS database (*n* = 9,692,841), regardless of health screening participation status; Table S3: Cross-sectional multivariable logistic regression model analyzing the association between irritable bowel syndrome and depression among all eligible individuals aged 19–64 years from the 2021 NHIS (*n* = 9,692,841), regardless of health screening participation status.

## Abbreviations

The following abbreviations are used in this manuscript:

IBS	Irritable Bowel Syndrome
NHIS	National Health Insurance Service
ICD-10	International Classification of Diseases, Tenth Revision
HPA	Hypothalamic–Pituitary–Adrenal
IRB	Institutional Review Board
CCI	Charlson Comorbidity Index
BMI	Body Mass Index
OR	Odds Ratio
CI	Confidence Interval
