# Supplementary material for: Depression Among Individuals with Irritable Bowel Syndrome: A Nationwide Claims-Based Analysis of 3.9 Million Koreans
Source: Healthcare (Basel). 2025 Nov 21;13(23):2998. doi: 10.3390/healthcare13232998 (PMC12691875; doi:10.3390/healthcare13232998)
Supplement: Supplementary file 1 [file healthcare-13-02998-s001.zip › healthcare-3862065-supplementary.pdf]

Table S1. Characteristics of the full eligible individuals aged 19-64 years from the 2021 NHIS database (N=9,692,841), regardless of their participation in health-screening exams

Category		Diagnosis of depression in 2021 N (%)		
		No depression (N=9,275,714)	Depression (N=417,127)	p-value
Age (mean±SD)		42.79±12.95	44.10±13.56	<.001
Sex	Male	4,799,574 (96.71)	163,503 (3.29)	<.001
	Female	4,476,140 (94.64)	253,624 (5.36)	
Residential area	Rural	4,136,889 (95.60)	190,618 (4.40)	<.001
	Urban	5,138,825 (95.78)	226,509 (4.22)	
CCI	0	6,513,976 (96.88)	209,555 (3.12)	<.001
	1	1,668,482 (94.12)	104,147 (5.88)	
	Over 2	1,093,256 (91.36)	103,425 (8.64)	

* NHIS, National Health Insurance Service; CCI, Charlson Comorbidity Index;

Table S2. One-year prevalence of depression among the full eligible individuals aged 19-64 years from the 2021 NHIS database (N=9,692,841), regardless of the health-screening participation status

		Depression diagnosis in 2021		Total N (%)	p-value
		None N (%)	Yes N (%)		
IBS diagnosis in 2021	None	8,606,844 (96.04)	354,956 (3.96)	8,961,800 (100.00)	<.001
	Yes	668,870 (91.50)	62,171 (8.50)	731,041 (100.00)	
Total		9,275,714 (95.7)	417,127 (4.3)	9,692,841 (100.00)	

* NHIS, National Health Insurance Service; IBS, irritable bowel syndrome

Table S3. Cross-sectional multivariable logistic regression model analyzing the association between irritable bowel syndrome and depression among the full eligible individuals aged 19-64 years from the 2021 NHIS (N=9,692,841), regardless of the health-screening participation status

		OR (95% CI)
Variables	Values	Model 1
IBS	Yes	1.82 (1.81, 1.84)
	No	Reference
Age (years)		1.00 (1.00, 1.00)
Sex	Female	1.61 (1.60, 1.62)
	Male	Reference
Residential Area	Urban	0.96 (0.96, 0.97)
	Rural	Reference
CCI	2+	2.86 (2.84, 2.89)
	1	1.89 (1.87, 1.90)
	0	Reference

* NHIS, National Health Insurance Service; CCI, Charlson Comorbidity Index
